# Socio-demographic characteristics and pharmacological treatment options in patients with delirium

**DOI:** 10.1192/j.eurpsy.2024.757

**Published:** 2024-08-27

**Authors:** F. J. Cruz Aviña, A. Salazar Rodriguez, D. N. M. Sanchez, E. A. C. Martinez, L. C. Rocha Reza, S. V. Nuñez Pichardo, H. A. Barranco Rogel, M. G. Ochoa Madrigal, O. Meneses Luna

**Affiliations:** ^1^Psychiatry, psychology and neuro psychology, Centro Médico Nacional 20 de noviembre; ^2^Psychiatry, Hospital Psiquiatrico Fray Bernardino Alvarez, Mexico City, Mexico

## Abstract

**Introduction:**

Delirium is common in hospital settings, with approximately 3% to 45% of older patients in hospitals developing delirium during their stay. Among the elderly and those with severe or advanced medical conditions, the reported percentage of patients with delirium is over 56%. The three motor subtypes of delirium are hyperactive, hypoactive, and mixed. Another way to characterize delirium is based on whether it is reversible, irreversible, or terminal.

**Objectives:**

Identifying appropriate pharmacological treatment options among antipsychotics and their correlation with various precipitating and predisposing factors in the in-hospital context

**Methods:**

This was a retrospective, cross-sectional, observational study that utilized a database created by the psychiatry department at the National Medical Center 20 de Noviembre, with data collected from April 2021 to April 2022. The database contains anonymized administrative and clinical data of patients who were seen in the psychiatry department for the diagnosis of any type of delirium, using the CAM scale for classification. The database includes records and data of hospitalized patients, encompassing all specialties at this medical center

**Results:**

A total of 139 patients were included in the study, of which 39% were female and 61% were male, with a mean age of 67 and a median age of 68 years. It was observed that the average duration of delirium symptoms, from receiving the consultation to remission, was approximately 6 days (p <0.005) (OR 5.12-6.62), and the average length of hospital stay was approximately 20 days (OR 17.3-22.09). Among the patients, 50.39% were overweight, 63% had hypertension (HTA), 29% had chronic kidney injury, 24% had a history of delirium, and 73% had recent surgical interventions. Patients with diabetes mellitus had a 3.1 times higher risk, those with HTA had a 2.8 times higher risk, and those with kidney injury had a 3.8 times higher risk of having a positive CAM result. It was observed that haloperidol, used in 84% of the patients, showed the highest percentage reduction in CAM scores

**Image:**

**Figure:**
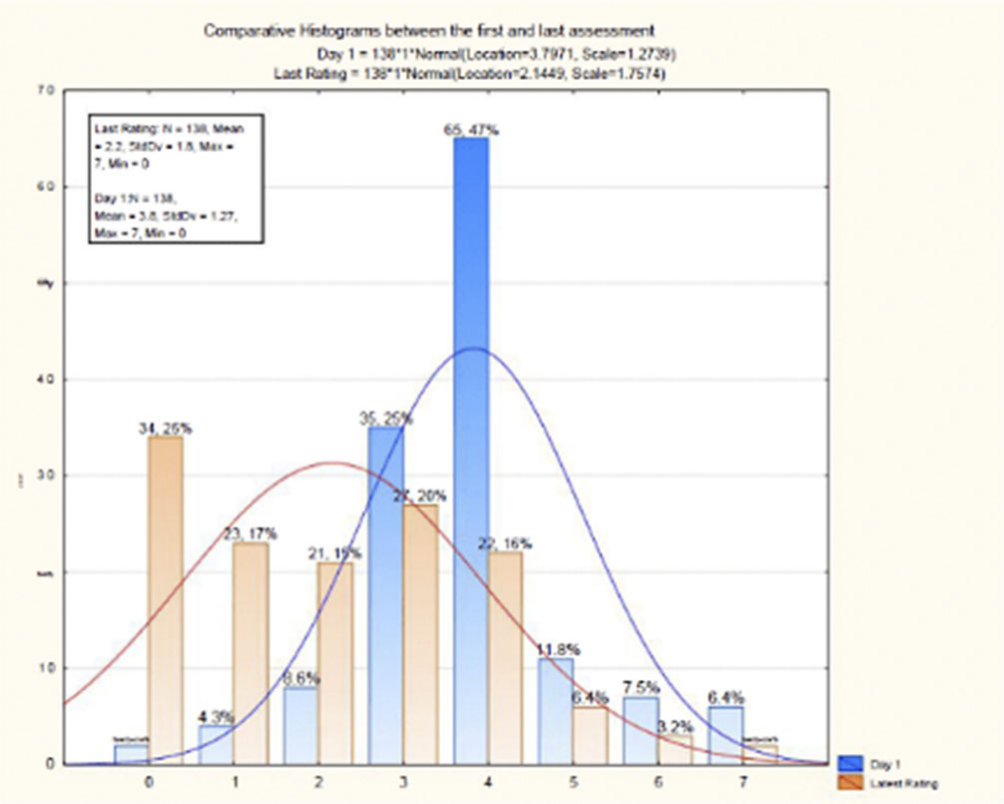

**Image 2:**

**Figure:**
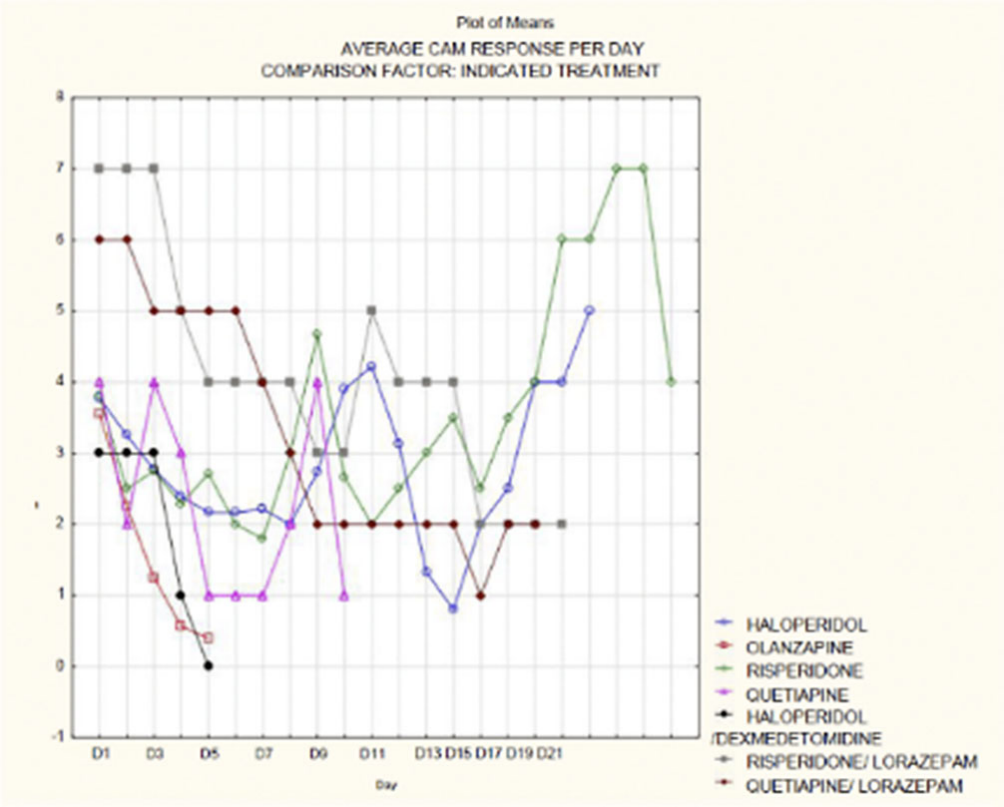

**Conclusions:**

The results of this study emphasize the importance of identifying risk factors associated with delirium and implementing effective treatment for this condition. It was observed that the average duration of delirium symptoms was approximately 6 days, which is relevant for understanding the course and management of this illness. Furthermore, it was found that the average hospital stay was 20 days, underscoring the burden that delirium can place on healthcare systems.

In conclusion, this study highlights the importance of identifying risk factors and providing appropriate treatment, such as the use of haloperidol, to improve outcomes in patients with delirium.

**Disclosure of Interest:**

None Declared

